# Advances in Synthesis and Applications of Single-Atom Catalysts for Metal Oxide-Based Gas Sensors

**DOI:** 10.3390/ma17091970

**Published:** 2024-04-24

**Authors:** Yuanting Yu, Yiling Tan, Wen Niu, Shili Zhao, Jiongyue Hao, Yijie Shi, Yingchun Dong, Hangyu Liu, Chun Huang, Chao Gao, Peng Zhang, Yuhong Wu, Linggao Zeng, Bingsheng Du, Yong He

**Affiliations:** 1 Key Laboratory of Optoelectronic Technology and Systems of the Education Ministry of China, College of Optoelectronic Engineering, Chongqing University, Chongqing 400044, China; 202208131049t@stu.cqu.edu.cn (Y.Y.); tan_yiling@163.com (Y.T.); chevron325@163.com (W.N.); zsl18678631330@163.com (S.Z.); 20230801031@stu.cqu.edu.cn (J.H.); 20172535@cqu.edu.cn (Y.S.); 202208021016@stu.cqu.edu.cn (Y.D.); 202308021039@stu.cqu.edu.cn (H.L.); 20192547@cqu.edu.cn (C.H.); gaoc@cqu.edu.cn (C.G.); 2 Chongqing Key Laboratory of Toxic and Drug Analysis, Chongqing Police College, Chongqing 401331, China; 13246827609@163.com (P.Z.); d20240677890@163.com (Y.W.); 3 Chongqing Institute for Food and Drug Control, Chongqing 401121, China; zenglinggao@cqifdc.org.cn; 4 Chongqing Key Laboratory of Optical Fiber Sensor and Photoelectric Detection, Chongqing University of Technology, Chongqing 400054, China

**Keywords:** single-atom catalysts, gas sensor, metal oxide, defect engineering, materials characterization

## Abstract

As a stable, low-cost, environment-friendly, and gas-sensitive material, semiconductor metal oxides have been widely used for gas sensing. In the past few years, single-atom catalysts (SACs) have gained increasing attention in the field of gas sensing with the advantages of maximized atomic utilization and unique electronic and chemical properties and have successfully been applied to enhance the detection sensitivity and selectivity of metal oxide gas sensors. However, the application of SACs in gas sensors is still in its infancy. Herein, we critically review the recent advances and current status of single-atom catalysts in metal oxide gas sensors, providing some suggestions for the development of this field. The synthesis methods and characterization techniques of SAC-modified metal oxides are summarized. The interactions between SACs and metal oxides are crucial for the stable loading of single-atom catalysts and for improving gas-sensitive performance. Then, the current application progress of various SACs (Au, Pt, Cu, Ni, etc.) in metal oxide gas sensors is introduced. Finally, the challenges and perspectives of SACs in metal oxide gas sensors are presented.

## 1. Introduction

Gas sensors are widely used in environmental protection [1,2,3], healthcare [4,5,6], food testing [7,8,9,10], agricultural production [11,12,13], and other fields. The development of the Internet of Things (IoTs) puts higher requirements on gas sensors with lower power consumption and higher performance. There are many types of gas sensors, including electrochemical, solid electrolyte, semiconductor, etc. Among them, chemiresistive gas sensors are the most widely used, with the advantages of low cost, good response, and ease of mass production. The core of chemiresistive gas sensors is the sensitive material, and metal oxides are the first commercially available and most researched sensitive materials, with the advantages of low cost, simple structure, and high stability. However, metal oxide-based gas sensors have the disadvantages of high operating temperature and poor selectivity [14].

To facilitate the disadvantages of metal oxide-sensitive materials, defect engineering [15,16,17], heterogeneous constructing [18,19,20,21], noble metal modification [22,23,24], and various other strategies have been used to improve the gas-sensitive properties of metal oxides. Among them, the strategy of noble metal modification uses the catalytic properties of noble metals to modify the sensitive materials in such a way that by modulating the adsorption properties and reactivity of gases on the surface of the materials, the enhancement of the selectivity of the sensors can be achieved, as well as the lowering of the operating temperature. For example, Behnam Bahrami et al. doped gold (Au) particles in SnO_2_ and investigated its selectivity in a model of a liquefied petroleum gas system (CO, methane, and propane), and they found that the introduction of Au particles improves the selectivity of SnO_2_ for CO and reduces the optimal operating temperature [25]. However, these catalysts of different sizes and morphologies will generate multiple active sites, resulting in the actual response of the sensor often being the “superposition effect” of these multiple catalytic effects, which results in a response to different types of gases and a decrease in their selectivity.

Single-atom catalysts are isolated single atoms dispersed on supports without any form of interaction between each individual atom [26,27,28]. It has been found that when doping or modifying the support material using single atoms, on the one hand, due to strong metal–support interactions, it promotes charge redistribution at the interface, which makes the support interface around the single atoms more active and more likely to adsorb gas molecules and promotes the chemical reaction at the gas/solid interface to increase the reactivity [29,30]. On the other hand, unlike heterogeneous catalysis produced by nano-catalysts, due to the single active site of single atoms, each active center will produce the same geometry, which will avoid the atomistic catalytic pathway, reduce the side reactions, and improve the reaction selectivity [31]. For example, Georgios Kyriakou’s group found that a single isolated Pd atom on the copper surface greatly reduces the energy barrier for hydrogen absorption and subsequent desorption on the copper metal surface and that hydrogen dissociation at the Pd atomic site and weak binding to copper resulted in excellent selectivity for the hydrogenation of styrene and acetylene compared to pure copper or palladium metal [32]. Based on those advantages of SACs, it has been successfully applied to make up for the shortcomings of metal oxide-sensitive materials. In recent years, the noble metal single-atom catalysts of Pt, Pd, Au, etc., have been commonly used for decorating the metal oxide gas sensing materials of SnO_2_, WO_3_, ZnO, and In_2_O_3_ to improve the response and selectivity or reduce the operation temperature.

However, research on the gas-sensitive properties of metal oxide-loaded SACs is still in its infancy [33,34]. There are some limitations that restrict its development, such as the limited synthesis and characterization methods, agglomeration and growth up of catalysts, and the unclear relationship between sensing performance and the catalysts. Thus, it is necessary to summarize the current synthesis and characterization methods, stabilization strategies, and the gas sensing properties in order to gain an insightful understanding of their development direction. In this review, we take the research progress of single-atom catalyst-functionalized metal oxide gas sensors as a starting point and systematically outline the synthesis and characterization methods of SACs, the interactions between SACs and metal oxide supports, as well as the related gas sensing performance studies. Finally, the challenges and perspectives of SACs in the application of metal oxide-based gas-sensitive materials are presented.

## 2. Synthesis Methods of SAC-Functionalized Metal Oxides

The synthesis of metal oxides loaded with SACs is a prerequisite for studying their structural features and exploring their applications. Achieving the monodisperse without the agglomeration of single-atom catalyst-modified metal oxides is the goal of exploring the synthesis strategies. Currently, the common strategies for loading SACs on metal oxides include atomic layer deposition, impregnation, co-precipitation, photochemistry, and space-limiting strategies. Next, we summarize several typical methods of SACs synthesis.

### 2.1. Atomic Layer Deposition (ALD)

**Figure 1:**
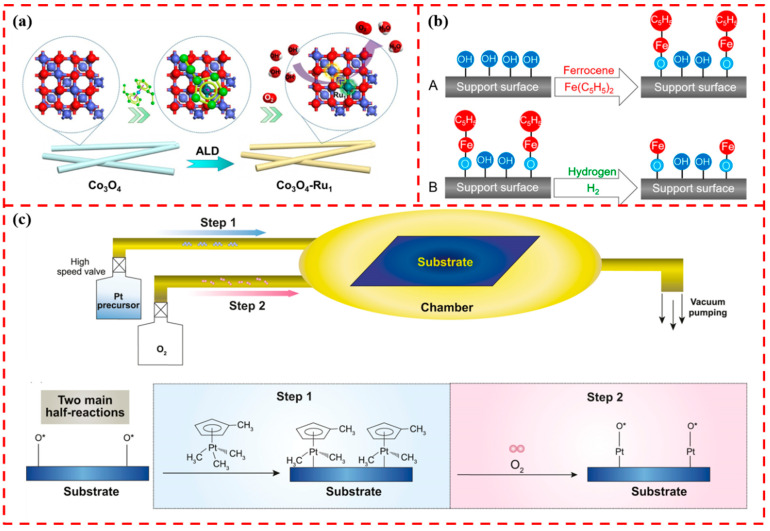
(**a**) Schematic representation of single-atom Ru loading on Co_3_O_4_ by ALD. Reprinted with permission from Ref. [38]. Copyright 2020, Elsevier. (**b**) Reaction scheme of the Fe-ALD half-reaction. Reprinted with permission from Ref. [39]. Copyright 2020, American Chemical Society. (**c**) Schematic representation of the deposition of Pt single atom and the two main half-reactions on a support by ALD. Reprinted with permission from Ref. [40]. Copyright 2018, Science Press.

Atomic layer deposition is a thin-film deposition technique based on a self-limiting surface reaction with high homogeneity. It is a technique that can be precisely controlled at the atomic level [35,36,37]. Numerous studies have been conducted to design and successfully synthesize SAC-loaded materials by the ALD method. The study of metal oxides as supports and loading SACs on their surfaces by ALD has been applied in the fields of CO oxidation, oxygen precipitation catalysis, gas sensing, etc. Zhao et al. succeeded in uniformly depositing Ru single atoms on the surface of Co_3_O_4_ by ALD, and the as-made Ru single atom served as a catalyst for the oxygen precipitation reaction, as shown in Figure 1a. The catalytic activity was increased to 95.5 times compared with that of pure Co_3_O_4_. Theoretical calculations showed that the Ru single atom acts as a promoter and improves the catalytic activity by modulating the binding energy between the intermediate and the active site [38]. Wang et al. reported a general ALD strategy with uniformly dispersed Fe single atoms loaded up to 1.78 wt% on multiwalled carbon nanotubes, SiO_2_, and TiO_2_ substrates, and the reaction scheme is shown in Figure 1b. Among them, the Fe/TiO_2_ system was used for the catalytic degradation of a methylene blue (MB) solution. Experimental investigations showed that the photocatalytic activity of TiO_2_ obtained after two Fe ALD cycle depositions was about six times higher than that of pure TiO_2_ [39]. Figure 1c illustrates the flowchart for the deposition of single-atom Pt on graphene. The Pt precursor first reacts with adsorbed oxygen (O*) on the graphene surface, and then the Pt precursor is converted to Pt-O species by oxygen pulses to form a new adsorbed oxygen layer on the Pt surface. The single-atom-loaded catalysts prepared by the ALD method showed excellent advantages, but clusters were easily formed because the metal atoms deposited by ALD preferred to be adsorbed on the existing metal atoms. Moreover, ALD technology is complicated to operate and requires expensive equipment, which limits its large-scale production application [40].

### 2.2. Impregnation

The impregnation method first adsorbs the metal precursor on the surface of the support and then thermally decomposes the metal precursor by annealing and removing the ligand while enhancing the interaction of the single atoms with the support. Compared with the ALD method, the impregnation method has the advantages of simple operation and low cost and is widely used in industry for loading metal catalysts [41,42]. Zhang et al. successfully loaded atomically dispersed Pt on RuO_2_ by impregnation and confirmed the random presence of Pt single atoms on the surface of RuO_2_ by high-angle annular dark-field (HAADF). The catalytic activity of Pt_1_/RuO_2_ for methanol oxidation was investigated and found to be 15.3 times higher than that of the commercial catalyst Pt/C. Pt_1_/RuO_2_ also exhibited excellent catalytic stability [43].

**Figure 2:**
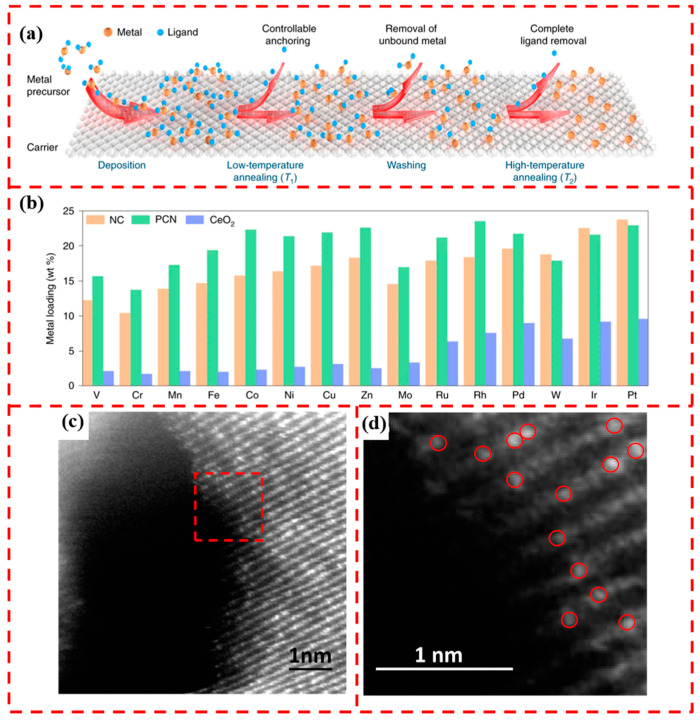
(**a**) Two-step annealing strategy for loading single atoms. (**b**) Metal loading achieved on NC, PCN, and CeO_2_ supports. Reprinted with permission from Ref. [45]. Copyright 2022, Springer Nature. (**c**) STEM-HAADF image of the Ni_x_Mg_1−x_O sample with Ni concentration of 10%. (**d**) Magnified image of the selected region of the red dotted square in (**c**), with Ni single atoms circled in red. Reprinted with permission from Ref. [47]. Copyright 2019, American Chemical Society.

The high surface free energy of single atoms makes them susceptible to migration and agglomeration, generating nanoclusters or nanoparticles with low surface energy [44]. The preparation of SACs by the impregnation method suffers from the disadvantages of low loading and non-uniform dispersion. Thus, it is necessary to inhibit atomic aggregation while increasing the single-atom loading rate. Hai et al. prepared SACs by combining impregnation method with the two-step annealing method, which is schematically shown in Figure 2a. Fifteen kinds of metals were successfully loaded on NC, PCN, and CeO_2_ supports with the highest loading of 23 wt% (Figure 2b), which greatly improved the single-atom loading rate and also provided an avenue for exploring the library of monometallic/multimetallic catalysts [45]. This is a novel extension of the impregnation method and demonstrates the potential of the impregnation method in achieving high SACs loads.

### 2.3. Coprecipitation and Hydrothermal

Coprecipitation is a typical method for the preparation of metal catalysts. In this method, uniformly loaded metal catalysts are prepared by using metal ions as precursors and adding a precipitant to an aqueous solution of the precursor to produce a precipitate [46]. Wei et al. obtained Pt single-atom-loaded FeO_x_ catalysts by coprecipitation using a chloroplatinic acid (H_2_PtCl_6_·6H_2_O) precursor and iron nitrate (Fe(NO)_3_·9H_2_O) aqueous solution. Due to the strong interaction between the Pt single atom and the FeO_x_ support, the catalyst acted for the hydrogenation of 3-nitrostyrene at a TOF of ~1500 h^−1^, with a selectivity of close to 99% for 3-aminostyrene, demonstrating excellent catalytic performance [31]. Millet et al. synthesized a series of Ni_x_Mg_1−x_O samples with different Ni concentrations (0–15 at%) by coprecipitation. Among them, the STEM-HAADF images of the samples with a Ni concentration of 10% are shown in Figure 2c. The Ni single-atom sites are marked with a red circle in Figure 2d, which serve as the active sites for CO_2_ activation [47].

Similarly, the hydrothermal method was adopted to synthesize SACs by adding the metal ions and salt solution to a stainless-steel autoclave reactor. For example, Shah et al. obtained Co-incorporated RuO_2_ catalysts by a one-pot hydrothermal process followed by calcination. The Co single atom not only greatly alleviated the stability problems of RuO_2_ under alkaline OER conditions but also substantially elevated the HER activity of Ru-based electrocatalysts under acidic HER conditions. [48]. Gu et al., through the modified hydrothermal method, prepared an Fe single-atom-decorated MnO_2_, which exhibited a stronger O_2_ activation performance than the conventional surface oxygen vacancy activation sites [49].

The coprecipitation and hydrothermal methods have certain disadvantages: the metal precursor and the support precursor are precipitated at the same time, and the metal catalyst will inevitably be embedded in the body material of the support, resulting in a certain degree of waste. However, coprecipitation and hydrothermal methods are easy to operate, low cost, and suitable for large-scale production [46]. At present, the application of the coprecipitation and hydrothermal methods for loading SACs occurs less often than that of the impregnation method in the wet chemical method, and it is worthwhile to further explore the application of this method.

### 2.4. Photochemical Method

The photochemical method does not require high-temperature pyrolysis compared to impregnation and coprecipitation. It is a mild method of loading SACs [50]. Liu et al. synthesized single-atom Pd-loaded TiO_2_ nanosheets via a photochemical route. TiO_2_ is inherently photoactive, and under UV irradiation, EG radicals are generated on the surface of TiO_2_. The presence of EG radicals helps in the removal of Cl^−^ from the Pd precursor ions, leaving Pd single atoms on the surface. In the hydrogenation of C=C bonds, the Pd_1_/TiO_2_ catalyst exhibits nine times higher catalytic activity than commercial Pd catalysts with excellent stability [51].

**Figure 3:**
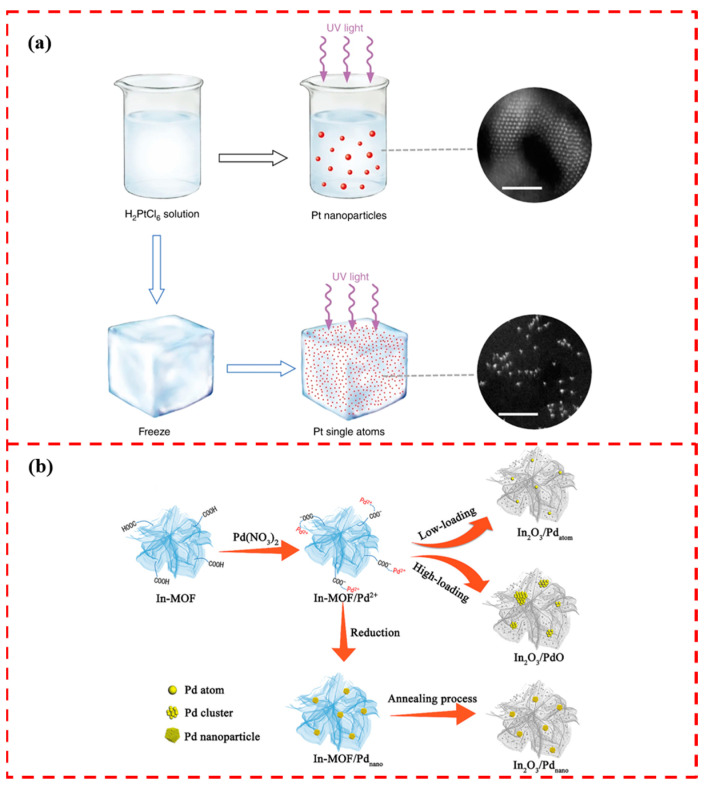
(**a**) Schematic diagram of cryo-photochemically loaded Pt single-atom catalyst. Reprinted with permission from Ref. [50]. Copyright 2017, Springer Nature. (**b**) Schematic diagram of In-MOF as a template for loading single-atom Pd. Reprinted with permission from Ref. [56]. Copyright 2021, John Wiley and Sons.

Wei et al. obtained atomically dispersed platinum by irradiating frozen chloroplatinic acid solutions with UV light. The ice lattice naturally restricts the migration and agglomeration of the atoms, and a flow chart of the method is shown in Figure 3a. Aberration-corrected high-angle annular dark-field scanning transmission electron microscopy (HAADF-STEM) characterization result plots confirm the successful loading of Pt single atoms. This method was applied to successfully loaded platinum single atoms on different supports such as mesoporous carbon, graphene, carbon nanotubes, titanium dioxide nanoparticles, and zinc oxide nanowires. The combination of low temperature and photochemistry is confirmed to be an effective method for loading single-atom catalysts. The photochemical method requires mild reaction conditions and has potential applications in the field of sustainable energy and green chemistry, contributing to the environmental friendliness of catalytic reactions [52].

### 2.5. Space Limitation Strategy

It is well known that metal–organic framework (MOF) materials are regularly porous. This feature facilitates the adsorption and anchoring of metal precursors, prevents agglomeration, and is more conducive to the successful synthesis of SACs [53]. MOFs can be pyrolyzed at high temperatures to obtain a series of derivatives of MOFs, such as nitrogen-doped porous carbon, metal oxides, etc., which tend to retain the porous characteristics of MOFs. Therefore, the spatial confinement of single atoms through MOF porous templates and subsequent high-temperature annealing steps to obtain single-atom-loaded MOF derivative materials is an effective strategy for loading SACs.

Currently, most studies focus on MOF-derived porous nitrogen-doped carbon materials loaded with SACs [54,55] and relatively few studies have been carried out on MOF-derived oxides loaded with single-atom metal catalysts. Liu et al. used In-MOF as a template, loaded with Pd single atoms, and after high-temperature pyrolysis treatment, Pd single-atom-modified In_2_O_3_ was obtained, and the preparation process is schematically shown in Figure 3b [56]. By varying the introduction amount of Pd precursor ions, Pd single-atom-loaded, PdO cluster-loaded In_2_O_3_ were synthesized. By annealing under a reducing atmosphere, Pd nanoparticle-loaded In_2_O_3_ was obtained. H_2_S gas was detected using the above three materials, in which the Pd single-atom-loaded In_2_O_3_ showed the optimal detection ability under the same testing conditions. This is attributed to the high uniformity of the loaded Pd single-atom sites, which greatly improves the surface adsorption energy for H_2_S gas. The study on MOF-derived oxides loaded with SACs deserves further investigation.

## 3. Characterization of SAC-Functionalized Metal Oxides

The successful loading of SACs on metal oxides needs to be demonstrated by corresponding material characterization techniques. Here, we summarize several typical characterization techniques that can directly prove the stable loading of SACs. These techniques can be divided into two main categories according to their results: (1) Morphological characterization: mainly including high-resolution high-angle annular dark-field scanning transmission electron microscopy (HAADF-STEM) and scanning tunneling microscopy (STM). (2) Structural information characterization: X-ray photoelectron spectroscopy, extended X-ray absorption fine structure, X-ray absorption near-edge structure, and Fourier-transform infrared spectroscopy.

### 3.1. HAADF-STEM and STM

**Figure 4:**
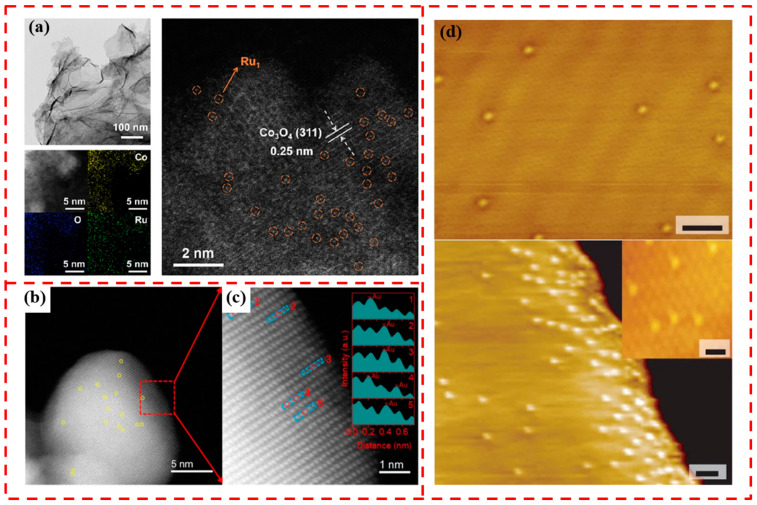
(**a**) TEM image of Ru_1_/CoO_x_, aberration-corrected HAADF-STEM EDS image and HAADF-STEM image of aberration-corrected Ru_1_/CoO_x_, where orange circles highlight monodisperse Ru atoms. Reprinted with permission from Ref. [59]. Copyright 2023, American Chemical Society. (**b**) High-resolution AC-HAADF-STEM image of Au_0.1_Ag_0.9_-ZnO. (**c**) Enlarged high-resolution AC-HAADF-STEM image of Au_0.1_Ag_0.9_-ZnO and the corresponding intensity profiles at the labelled positions. Reprinted with permission from Ref. [60]. Copyright 2023, John Wiley and Sons. (**d**) STM image of 0.01 ML Pt/Cu(111) SAA surface. Reprinted with permission from Ref. [61]. Copyright 2018, Springer Nature.

HAADF-STEM is a transmission electron microscopy technique that allows images with atomic-level resolution to be obtained by measuring the high-angle scattering of transmitted electrons in a sample. Compared to conventional TEM, the contrast of HAADF-STEM images correlates with atomic number, and the atoms of heavier elements produce more scattering than lighter elements, appearing brighter in the image, which can significantly improve the detection sensitivity at sizes below 1 nm. HAADF-STEM can provide the most intuitive evidence for the successful loading of SACs [57,58]. As shown in Figure 4a, which is the TEM image of CoO_x_ loaded with single-atom Ru, it can be observed that the initial morphology of CoO_x_ does not change after the introduction of single-atom Ru. EDS mapping confirmed that the Ru element was uniformly distributed in CoO_x_ nanosheets. Through HAADF-STEM images, some bright spots on the surface of CoO_x_ can be observed, which is atomically dispersed Ru, highlighted by orange circles in the Figure 4a [59]. In Figure 4b, several atom-sized bright spots (highlighted with yellow circles) can be clearly observed, which are attributed to the presence of Au single atoms in the Au_0.1_Ag_0.9_ alloy NPs. Figure 4c shows the intensity distribution of the labelled regions in Figure 4b, further confirming the atomic dispersion of Au in the Au_0.1_Ag_0.9_ alloy NPs [60].

STM is a scanning probe microscopy technique with atomic-level resolution, but because it cannot transmit the sample, it can only provide information about the sample surface. However, it also makes up for the shortcomings of HAADF-STEM in which the electron beam may cause electron radiation damage to the sample. Figure 4d shows the STM image of the Pt/Cu monoatomic alloy, and it can be clearly observed that in the Pt/Cu monoatomic alloys (SAAs), the Pt atoms are able to alloy directly into the area near the steps and step edges and are randomly distributed over the entire surface [61].

### 3.2. XANES and EXAFS

The isolated presence of single atoms can be visualized by HAADF-STEM and STM technique characterization, but the help of spectroscopic analysis techniques is required to further analyze the detailed chemical information of single atoms. X-ray absorption spectroscopy (XAS) plays an important role in probing the electronic structure, geometry, coordination environment, and other information of atoms. According to the division of energy spectra in different regions, the XAS technique can be divided into X-ray near-edge absorption fine structure spectroscopy (XANES) and X-ray extended-edge absorption fine structure (EXAFS). XANES measures the region from −50 to 150 eV away from the absorption edge, which is associated with the jump of electrons from the inner shell to valence orbitals. EXAFS measures the energy range from 150 to 1000 eV, and the local environment of the atoms in the material is obtained by analyzing the structure of oscillations away from the absorption edge in the X-ray absorption spectra. These characterization techniques can provide information about interatomic distances, coordination numbers, and atom types [62]. The application of XANES and EXAFS techniques can provide a more comprehensive understanding of the atomic structure of single-atom catalysts [63].

**Figure 5:**
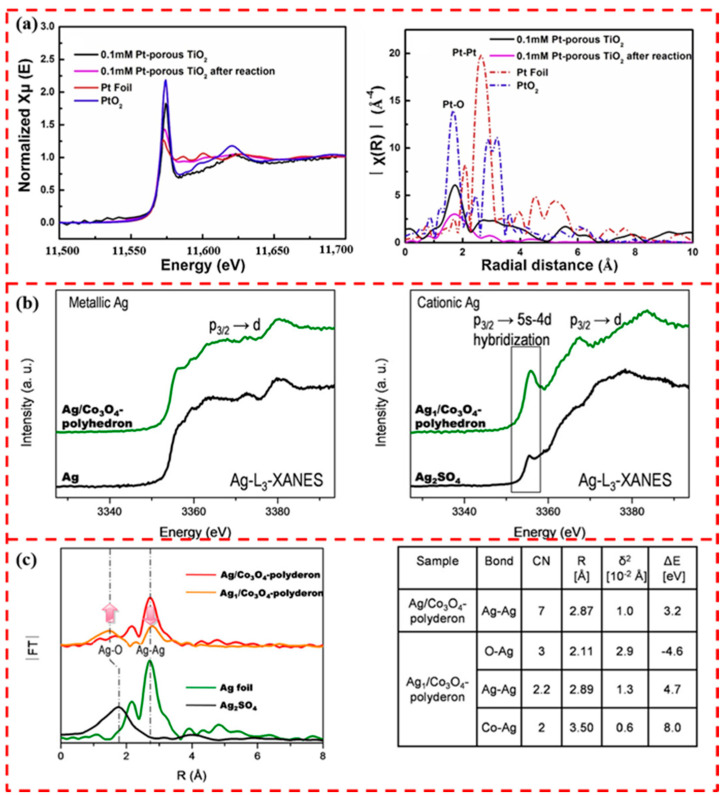
(**a**) Pt L3-edge XANES, and R-space FT-EXAFS spectra. Reprinted with permission from Ref. [64]. Copyright 2019, Elsevier. (**b**) Ag L3-edge XANES spectra of Ag/Co_3_O_4_ polyhedral samples, Ag foil standards, Ag_1_/Co_3_O_4_ polyhedral samples, Ag_2_ SO_4_ Ag L3 edge XANES spectra of polyhedral samples and Ag foil standards. (**c**) Fourier transform of EXAFS spectra of Ag_1_/Co_3_O_4_ polyhedron, Ag_1_/Co_3_O_4_ polyhedron with respect to Ag foil and Ag_2_SO_4_, and results of the fitting of Ag-K edge EXAFS data. Reprinted with permission from Ref. [65]. Copyright 2020, Wiley-VCH Verlag GmbH & CO. KGaA.

Xu et al. performed XANES and EXAFS analyses in order to investigate the chemical state and coordination environment of single-atom Pt in anchored non-porous TiO_2_ and obtained the spectra shown in Figure 5a. In Figure 5a, the intensity of the white line reflecting the oxidation state of Pt is between Pt foil and PtO_2,_ indicating that the valence state of Pt in the sample is between 0 and +4. In the R-space FT-EXAFS spectrum, only a prominent peak centered on the Pt-O contribution is observed at 1.68 Å, and almost no Pt-Pt contribution is observed, suggesting that only single Pt atoms are anchored in the non-porous TiO_2_, and no Pt nanoparticles are present [64]. Huang et al. prepared atomically dispersed Ag on polyhedral Co_3_O_4_ surfaces by top–down approach. The white line intensity of the samples obtained from Ag-L3-XANES measurements was compared with the standards. As shown in Figure 5b, the near-edge spectrum of the Ag/Co_3_O_4_ -polyhedron fresh sample is very similar to that of Ag metal. For metallic Ag, the white line adsorption at about 3355 eV is relatively weak, and it can be determined that the Ag in the sample is mainly in a non-metallic state. In contrast, the white line intensity of the Ag_1_/Co_3_O_4_ -polyhedron sample is similar to that of Ag_2_SO_4_, indicating that Ag cations dominate in the thermally aged sample. Figure 5c shows the Ag K-edge extended XAFS (EXAFS) analysis of Ag_1_/Co_3_O_4_ and Ag/Co_3_O_4_. The Ag-Kedge spectra of Ag/Co_3_O_4_ polyhedron samples and the Ag-Kedge spectra of Ag foils are not significantly different except for the decrease in amplitude, which suggests that the Ag atoms in the Ag/Co_3_O_4_ polyhedron sample are mainly retained in the metal. The Ag atoms of the polyhedral samples are mainly retained the original Ag lattice of the metal particles. After calcination at 250 °C, another tunable peak can be seen in the EXAFS in the 1 to 2 Å region of the Ag_1_/Co_3_O_4_ polyhedron, which is attached to Ag-O. This indicates that the surface Ag atoms are oxidized in agreement with the XANES findings. Fitting the EXAFS data to this region, as shown in Figure 5c, yields atomically dispersed Ag cations with three Ag-O bonds [65].

### 3.3. FT-IR

Different molecules can absorb infrared radiation in a specific range, causing the chemical bonds within the molecules to vibrate, rotate, or stretch, resulting in a specific vibrational frequency. Infrared spectroscopy techniques can be used to analyze and identify compounds based on this principle. Infrared techniques are widely used in the characterization of catalysts due to their excellent sensitivity and the affordability of the equipment [66,67]. Infrared spectroscopy can indirectly obtain molecular information by tracking the adsorption of the probe molecule on the catalyst surface. Using CO as the probe molecule, the frequency shift of the C–O vibration can be analyzed to obtain the metal type, adsorption site, and other related information at the atomic level, which can provide rich information about the structure of the SACs [68,69,70]. Fourier-transform infrared spectroscopy (FT-IR) is an effective technique for the characterization of SACs, which uses the Fourier-transform technique to simultaneously acquire information over the entire infrared spectral range. This improves the speed of data acquisition and signal-to-noise ratio compared to conventional dispersive infrared spectrometers.

**Figure 6:**
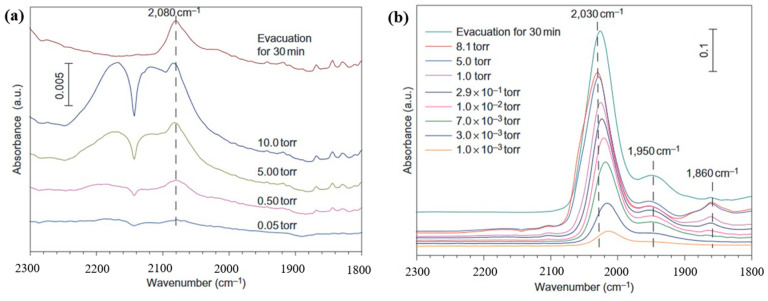
(**a**) In situ FT-IR spectrum of Pt-0.17 wt%-FeOx (sample A, FeOx with a Pt loading of 0.17 wt%). (**b**) In situ FT-IR spectrum of Pt-2.5 wt%-FeOx (sample B, FeOx with a Pt loading of 2.5 wt%). Reprinted with permission from Ref. [71]. Copyright 2011, Springer Nature.

Qiao et al. prepared FeO_x_ with a Pt loading of 0.17 wt% and FeO_x_ with a Pt loading of 2.5 wt%, which are noted as Sample A and Sample B, respectively. The FT-IR spectra of the two samples are shown in Figure 6a,b, respectively [71]. In the in situ FT-IR spectrum of sample A, CO adsorption shows only one weak band at 2080 cm^−1^, which is attributed to Pt^δ+^. In the in situ FT-IR spectrum of sample B, a strong vibrational and two weak vibrational bands are generated at 2030 cm^−1^, 1860 cm^−1^, and 1950 cm^−1^, respectively. The strong vibrational band at 2030 cm^−1^ is attributed to the linear bonding of the CO on the Pt^0^ site. The bands at 1860 cm^−1^ and 1950 cm^−1^ are generated at two Pt atoms bridging adsorbed CO and adsorbed CO at the direct interface between the Pt cluster and the carrier. Different to Figure 6b, the energy band positions of CO adsorbed in sample A in Figure 6a hardly change with the increase in CO pressure, which is caused by the lack of interaction force between CO molecules due to the atomic dispersion of Pt on the surface of sample A. The FT-IR spectra confirm that sample A contains only positively charged Pt single atoms, while sample B contains both Pt single atoms and clusters.

## 4. Stabilization Strategies of Single Atoms in Metal Oxides

Due to the high surface energy, single atoms tend to migrate and thus agglomerate on the support surface. How to maintain the stability of single atoms on the surface of metal oxides is the primary condition for the preparation of SAC-functionalized metal oxides [72,73]. The interaction between the single atoms and the substrate oxide is what ensures its stability [74]. In 1978, Tauster et al. first introduced the concept of strong metal–support interactions by investigating the reasons for the changes in the adsorption capacity of TiO_2_ loaded with noble metal nanoparticles for hydrogen and CO before and after high-temperature reductions and demonstrated that it is such strong metal–support interactions that lead to changes in the adsorption properties of metals [75]. In 2020, Han et al. found that TiO_2_ nanosheets loaded with single-atom Pt also showed the complete inhibition of CO adsorption at higher reduction temperatures. This is the interaction between the single-atom Pt and TiO_2_ that saturates the Pt atoms with coordination, confirming, for the first time, the existence of strong metal–support interactions in single-atom-loaded substrate materials [76]. The stronger the interaction between the single atom and the oxide substrate, the less likely the single atom is to migrate, which would thus lead to agglomeration. Therefore, enhancing the interaction between the single atom and the oxide substrate is the key to improving the stability of the single atom. Currently, the commonly used strategies are mainly to enhance the interaction by constructing defects on the support surface, introducing oxygen-containing functional groups and providing spatial confinement through the microporous structure of the support.

### 4.1. Stabilization through Defects

**Figure 7:**
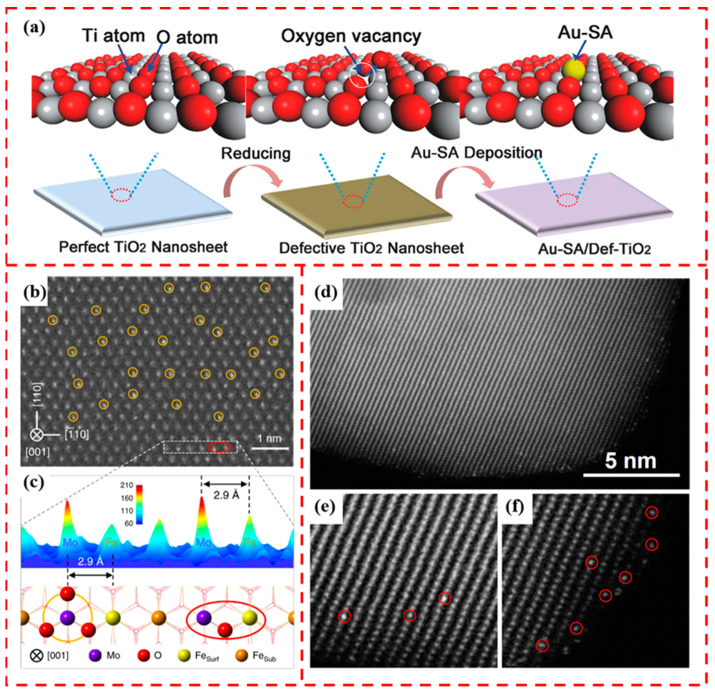
(**a**) Schematic diagram of Au single atoms anchored at oxygen vacancies on the surface of TiO_2_. Reprinted with permission from Ref. [82]. Copyright 2018, Wiley-VCH Verlag GmbH. (**b**) AC-STEM image of Mo_1_/Fe_2_O_3_, where Mo single-atoms are marked with yellow circles. (**c**) Intensity surface map and structural model of the selected region in (**b**) (white dashed rectangles). Reprinted with permission from Ref. [85]. Copyright 2020, Springer Nature. (**d**) Typical high-resolution HAADF-STEM image of 0.05Pt_1_/Fe_2_O_3_. (**e**) Pt single atoms on the surface of Fe_2_O_3_ are observed as bright spots. (**f**) Pt single atoms on the surface of Fe_2_O_3_ are occupied by Fe atoms, marked by red circles. Reprinted with permission from Ref. [79]. Copyright 2018, IOP PUBLISHING LTD.

Reducible metal oxide surfaces are prone to oxygen vacancies [77,78] or metal cations vacancies [79,80], which is an effective strategy for anchoring single atoms. Sanchez and Gazquez proposed a generalized model in which metal atoms interact strongly through lattice vacancies with oxygen ions in metal oxide supports. This strong interaction makes metal atoms nested at surface defects more stable than those on ideal surfaces, which inhibits the sintering of metal atoms under harsh reaction conditions [81]. Wan et al. anchored Pt single atoms at oxygen vacancies on the TiO_2_ surface to limit the migration through Ti-Pt strong interactions. The oxygen vacancies were used to stabilize the Au single-atom sites by constructing a Ti-Au-Ti structure while lowering the energy barrier and eliminating the competitive adsorption to improve the catalytic performance. The formation is schematically demonstrated in Figure 7a [82]. In addition, bimetallic active sites can be constructed by utilizing the oxygen vacancies on the surface of metal oxides, Zhang et al. successfully anchored Rh single atoms at the oxygen vacancies on the surface of Co_3_O_4_. In this case, Rh binds to three Co atoms, resulting in the formation of Rh_1_Co_3_ bimetallic sites, which is an effective way to develop efficient catalysts [83]. Metal cation vacancies are also common defects on the surface of reducible metal oxides [84]. Qu et al. exploited the formation of triple hollow sites formed by three surface lattice oxygen atoms on the surface of Fe_2_O_3_ (001) due to the absence of Fe ions. A single Mo ion occupies such a hollow site. It forms a binuclear site with the neighboring Fe ion (Figure 7b,c), providing a design strategy for developing improved selective catalytic reduction catalysts [85]. As demonstrated by DFT calculations, the Fe cation site is the most stable occupation site for Pt atoms on the 001 surface of Fe_2_O_3_. As shown in Figure 7d–f, the Pt single atoms all occupy the positions of Fe atoms [79].

For irreducible metal oxides, the coordination of unsaturated centers on their surfaces also provides effective sites for anchoring metal atoms. Kwak et al. proposed that the coordination of unsaturated penta-coordination Al^3+^ (Al^3+^ _penta_) on the (100) surface of the g-Al_2_O_3_ surface is the driving force for strong metal–support interactions. At low Pt loading (<1 wt%), the anchoring of Pt atoms through this coordinated unsaturated site forms the atomic dispersion of Pt on the Al_2_O_3_ surface [86].

### 4.2. Stabilization through Surface-O(OH)_x_ Species

Anchoring metal single atoms by –O or –OH on the oxide surface is an effective way to load SACs, whether for reducible or non-reducible supports [87,88,89]. Yang et al. found that the noble metals Au can form –O and –OH bonds with a variety of alkali ions, which in turn lead to the formation of stable single-dot cations Au–O(OH)_x_ species. In the catalytic reaction of water–gas shifts, the loaded single-atom Au species have the same catalytic activity for non-reducible carriers such as KLTL zeolite and mesoporous MCM-41 silica and reducible carriers such as cerium dioxide and iron oxide [90]. It was further found that for the three types of supports, TiO_2_, KLTL zeolite, and mesoporous MCM-41 silica, the Pt catalysts without added Na ions existed in large quantities as nanoparticles on all three types of supports. In contrast, more than 80% of the Pt catalysts added with Na ions existed in the form of isolated atoms and did not contain nanoparticles. The addition of Na ions increases the amount of hydroxyl radicals on the surface, which makes it easier for the formation of Pt–O(OH)_x_ active species [91]. Wang et al. revealed that the terminal hydroxyl groups on the surface of γ-Al_2_O_3_ are essential for anchoring Ag single atoms. The presence of more abundant terminal hydroxyl groups on the nanoscale γ-Al_2_O_3_ compared to the microscale γ-Al_2_O_3_ allows the Ag catalyst to form atomic dispersion instead of clusters, leading to higher catalytic performance [89]. The use of –O(OH)x species to achieve anchoring to metal atoms is a more widely applicable method for loading SACs.

### 4.3. Stabilization through Spatial Constraints

**Figure 8:**
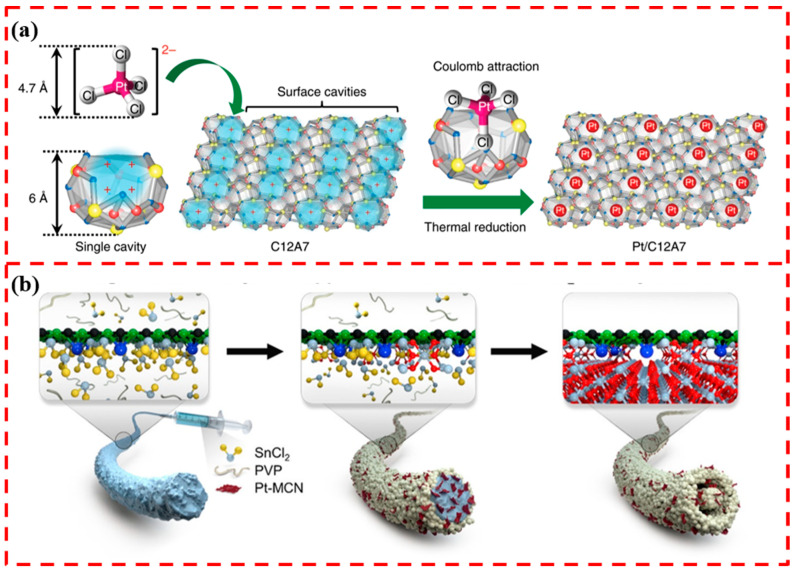
(**a**) Schematic representation of the positively charged surface cavity of C12A7 stabilizing a single Pt atom. Reprinted with permission from Ref. [99]. Copyright 2020, Springer Nature. (**b**) Pt single-atom catalyst stabilized at the carbon nitride/SnO_2_ heterojunction. Reprinted with permission from Ref. [100]. Copyright 2020, American Chemical Society.

The rational design of the internal pores of a material is also a strategy to stabilize single-atom catalysts. The spatial confinement of individual atoms by using pores can inhibit SAC sintering [92,93,94]. Carbon-based materials and zeolite series materials are characterized by their porous nature and are widely used as supports for anchoring SACs [95,96,97]. In addition, there have also been some studies on synthesizing metal oxides with porous structures as supports for anchoring SACs [98]. Ye et al. synthesized 12CaO-7Al_2_O_3_ material (C12A7) with a unique cage-like structure. The cavity of this cage-like structure is positively charged and interacts with the metal anion to achieve metal anion separation. The cavity size of 0.4 nm is just suitable for accommodating single metal atoms (Figure 8a). The unique stabilizing effect of the cavity nanostructures on the Pt single atoms was demonstrated by theoretical simulations and experimental characterization, and they remained atomically dispersed at a high temperature of 600 °C [99]. Zhang et al. loaded Pt catalysts on the surface of Al_2_O_3_ materials with and without mesoporous structure and confirmed that the mesoporous structure and the unique inner surface skeleton play a decisive role in achieving the atomic dispersion of Pt by comparing the results of the material characterization [93].

The use of heterojunctions to achieve the spatial confinement of the SAC is also an effective method. Shin et al. used metal oxide nano-heterostructures to achieve the effective stabilization of Pt SACs (Figure 8b). The heterojunction composed of carbon nitride/SnO_2_ not only spatially confines the Pt single-atom catalyst but also participates in the catalysis of the surface reaction of formaldehyde gas detection, which greatly improves the catalytic performance of the SACs system [100]. Enhancing the interaction between SACs and metal oxide support through the spatial restriction of porous structures or heterojunctions can inhibit the sintering failure of SACs. However, there are still few studies on this aspect, and it is necessary to explore more kinds of metal oxide supports further to achieve the anchoring of SACs through spatial confinement.

## 5. Gas-Sensitive Properties of SAC-Functionalized Metal Oxides

At present, applications regarding metal oxide gas-sensitive materials functionalized with SACs are mainly focused on SnO_2_, WO_3_, ZnO, and In_2_O_3_. In addition, Fe_2_O_3_, TiO_2_, and Co_3_O_4_ functionalized by SACs have also been reported, but with fewer studies. The existing studies are discussed in detail in this section.

### 5.1. SnO_2_-Based Support

**Figure 9:**
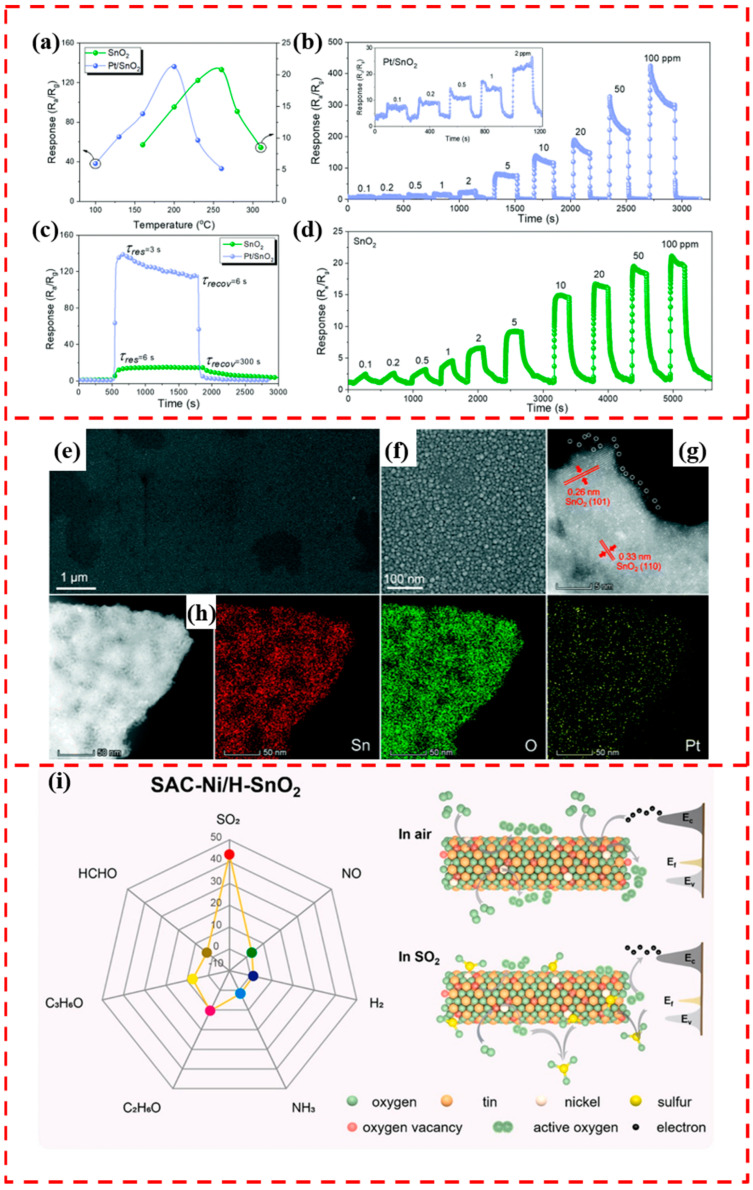
(**a**) Response of SnO_2_ and Pt/SnO_2_ films to 10 ppm TEA at different temperatures. (**b**) Response of Pt/SnO_2_ films to 0.1–100 ppm TEA at 200 °C. (**c**) Dynamic response of SnO_2_ and Pt/SnO_2_ films to 10 ppm TEA at 200 °C. (**d**) Response of SnO_2_ films to 0.1–100 ppm TEA at 200 °C. (**e**,**f**) SEM image of SnO_2_ film. (**g**) HAADF-STEM image. (**h**) EDS STEM elemental mapping of single-atom Pt/SnO_2_ film. Reprinted with permission from Ref. [107]. Copyright 2020, ROYAL SOC CHEMISTRY. (**i**) Schematic representation of the selectivity of SAC-Ni/H-SnO_2_ to different gases at 250 °C, 40% RH, and sensing of SO_2_. Reprinted with permission from Ref. [108]. Copyright 2022, Elsevier.

SnO_2_ is the first commercialized gas sensor, and it is one of the most studied metal oxide materials for its excellent gas-sensitive response, high stability, and low cost [101,102]. The surface modification of SnO_2_ by single atoms has developed a new potential for SnO_2_ as a gas-sensitive material [103,104,105,106]. In 2020, Xu et al. developed Pt single-atom-modified SnO_2_ thin-film sensitive materials, which is the first report of Pt single-atom-catalyst-modified SnO_2_ for gas-sensing applications [107]. The ALD technique was used to deposit SnO_2_ films on SnO_2_/Si wafers and Pt SACs on SnO_2_ films with trimethyl(methylcyclopentadienyl)platinum (IV) (MeCpPtMe_3_) (99%) and O_3_ as the precursors. Compared with the pristine SnO_2_, the Pt/SnO_2_-based sensor has a 60 °C lower optimal operating temperature (Figure 9a) and a response up to 136.2 for 10 ppm TEA, which is seven times higher than that of the pristine SnO_2_ (20.3). Meanwhile, Pt/SnO_2_ has a faster response/recovery time, reaching an ultra-high sensitivity of 8.76 ppm^−1^ and a very low limit of detection (LOD) of 7 ppb (Figure 9b–d). By SEM characterization, the structure of Pt/SnO_2_ was unchanged compared with the SnO_2_ film (Figure 9e,f). Brighter contrast images of Pt single atoms uniformly dispersed on SnO_2_ were obtained using high-angle annular dark-field scanning transmission electron microscopy (HAADF-STEM) (Figure 9g,h), confirming the successful loading of Pt single atoms. The single platinum atoms loaded on SnO_2_ have a chemical sensitization effect that dissociates atmospheric oxygen molecules into negatively charged oxygen ions, thereby increasing the adsorbed oxygen on the surface of tin dioxide. This feature improves the gas-sensitive properties of Pt/SnO_2_. In the meantime, the presence of single Pt atoms can reduce the activation energy of the surface reaction and enhance the adsorption and desorption of TEA. Zhou et al. prepared single-atom Rh-modified SnO_2_ nanoparticle-sensitive materials for formaldehyde detection by ALD [102]. The response of Rh/SnO_2_ to 20 ppm formaldehyde was 36.3, which was about 23 times higher than that of pure SnO_2_, and the response and recovery times were 4 s and 9 s, respectively. DFT theoretical calculations revealed that the reason for the improved gas-sensitive performance was that the Rh atoms increased the adsorption and charge transfer between formaldehyde molecules and SnO_2_.

In addition to noble metal SACs modification, non-precious metal single-atom modification on the surface of SnO_2_ also has excellent gas-sensitive response properties. Liu et al. loaded a Ni single atom on the surface of SnO_2_ by impregnation. K-edge X-ray absorption near-edge structure (XANES) results showed that the valence of Ni in SAC-Ni/SnO_2_ was at +2, and there were no observed Ni-Ni coordination-related FT-EXAFS peaks, which suggests that only single Ni atoms are present in SnO_2_, and no Ni nanoparticles are present [108]. The O_3_C vacancy and cavity SAC-Ni are candidate sites for capturing SO_2_. SAC-Ni adsorbs SO_2_ and then reacts with superoxide radicals generated from nearby oxygen vacancies to achieve the highly selective detection of SO_2_ (Figure 9i), demonstrating that the designed SAC-Ni/H- SnO_2_ sensor is reasonable and effective. The SAC-Ni/H-SnO_2_ sensor with Ni single-atom loading and enriched with oxygen vacancies showed a response value of 48 for 20 ppm SO_2_ and a detection limit of 100 ppb. DFT theoretical calculations showed that the oxygen vacancies and SAC-Ni are the candidate sites for the capture of SO_2_. Therefore, the gas-sensitive performance of SAC-Ni/H-SnO_2_ was substantially improved compared to SnO_2_.

Inorganic supports have the disadvantages of having a small area and a lack of stable defect sites compared to organic or organic–inorganic composite supports, resulting in relatively low single-atom catalyst loadings (<1 wt%). Shin et al. developed a general method for the preparation of SACs on metal and metal oxide supports by using N-doped graphene as a spatially confined single-atom sacrificial template [109]. Inductively coupled plasma atomic emission spectroscopy results showed successful loading up to 2.12 wt% of Pt single atoms on SnO_2_ nanosheets. The Pt-SA-functionalized Pt_1_-SnO_2_ NSs sensors exhibited superior sensitivity for acetone detection. For 10 ppm acetone, the response values of Pt_1_-SnO_2_ NSs were improved by 7.6-fold and 4.0-fold, respectively, compared to the original SnO_2_ NSs (R_air_/R_gas_ = 12.6) and Pt_n_-SnO_2_ (R_air_/R_gas_ = 23.8). SAC-functionalized SnO_2_ sensitive materials show surprising gas-sensitive properties. The selection of suitable single atoms for synergistic interactions with SnO_2_ provides a new strategy for the enhancement of gas-sensitive performance.

### 5.2. WO_x_-Based Support

**Figure 10:**
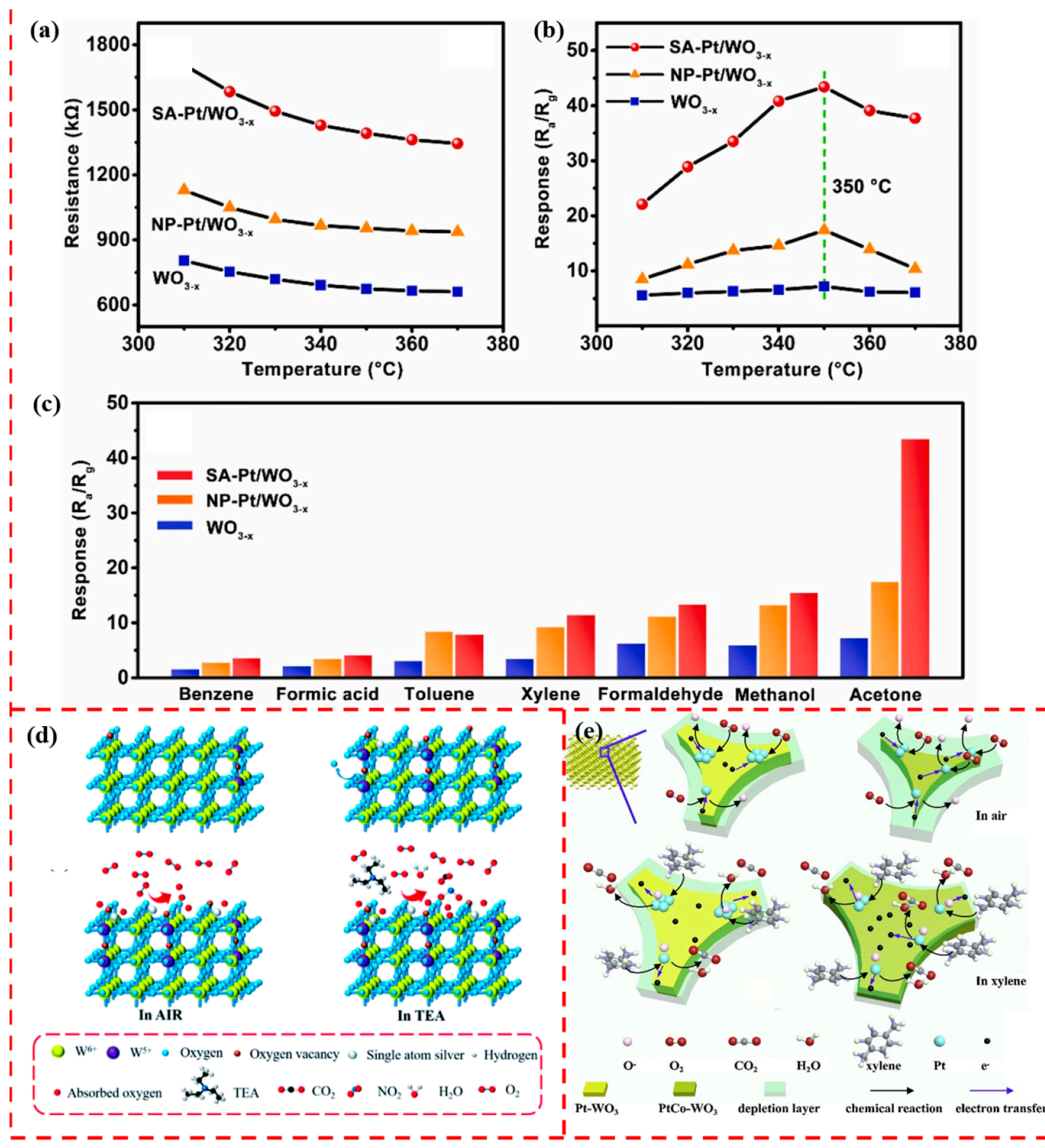
(**a**) Resistance change in air at different temperatures. (**b**) Response to 5 ppm acetone at different operating temperatures, which all reach optimal response at 350°C and (**c**) selectivity test for WO_3−x_, NP-Pt/WO_3−x_, and SA-Pt/WO_3−x_ for different vapors at 5 ppm (T = 350 °C, RH = 40%). Reprinted with permission from Ref. [112]. Copyright 2023, Elsevier. (**d**) Schematic representation of WO_3_ before and after loading with single-atom Ag and schematic representation of the reaction mechanism of single-atom Ag-loaded WO_3_ in air and TEA. Reprinted with permission from Ref. [113]. Copyright 2021, ROYAL SOC CHEMISTRY. (**e**) Sensing mechanism diagrams of Pt-WO_3_ and PtCo-WO_3_ samples. Reprinted with permission from Ref. [114]. Copyright 2019, Elsevier.

WO_3_ is an n-type semiconductor with a low bandgap (2.8 eV), which has the advantages of a superior charge transfer capability, good stability, and easy preparation. Therefore, WO_3_ has gradually become a popular gas-sensitive material. However, the performance of pure WO_3_ cannot fully meet the needs of practical applications, and the modification of its surface by adding noble metals is a way to improve the gas sensing performance [110]. Gu et al. prepared three kinds of sensitive materials, pure WO_3_, single-atom Pt-loaded WO_3_, and Pt nanocluster-loaded WO_3,_ and designed gas sensors, denoted as WO_3_, SA-Pt/WO_3_, and NC-Pt/WO_3_, for the detection of triethylamine and trimethylamine gases produced by fish spoilage [111]. The optimal operating temperature of the SA-Pt/WO_3_ gas sensors was reduced by 20 °C compared to WO_3_. Although the Pt loading in NC-Pt/WO_3_ is higher than that in SA-Pt/WO_3_, SA-Pt/WO_3_ has the best TEA detection among the three, with a sensitivity of 28.37 ppm^−1^ and a detection limit of 0.18 ppb. This is because of the atomic dispersion results in the exposure of every Pt atom and the formation of reactive sites, which leads to a higher adsorbed oxygen content in SA-Pt/WO_3_ than in NC-Pt/WO_3._ The superior TEA detection properties of SA-Pt/WO_3_ were attributed to the high adsorbed oxygen content and low activation energy. Yuan et al. loaded Pt single atoms and Pt nanoparticles on the surface of WO_3−x_, which is rich in oxygen vacancies. The surface atoms of the nanoparticle Pt have many coordination environments compared to single Pt atoms, while the individual atoms exist in similar chemical states. The SA-Pt/WO_3−x_ gas sensor not only has a higher response compared to Pt nanoparticle-loaded WO_3−x_ (Figure 10a,b) but also exhibits a high selectivity for acetone (Figure 10c) [112].

The synergistic interaction of rationally designed noble metal single atoms with oxygen vacancies or transition metal atoms on the surface of WO_3_ has significant advantages for enhancing gas-sensitive properties. It has been shown that Ag single atoms deposited on the surface of WO_3_ promote the formation of incomplete valence W^5+^, which increases the oxygen vacancy content [113]. The Ag single atoms on the surface of WO_3_ act as active sites, lowering the energy barrier and improving the adsorption performance of the target gas. Oxygen vacancies as active sites increased the rate of oxygen adsorption (Figure 10d). Therefore, at the most suitable Ag loading, the Ag-WO_3_-2 (pure WO_3_ under 0.01 mol L^−1^ silver nitrate solution), detected via a low-temperature deposition sensor, exhibited a superior gas-sensitive performance with a 5150 response for 50 ppm TEA target gas at 175 °C, a detection limit of 1.7 ppb, and long-term stability as well as high selectivity and reproducibility. Gu et al. synthesized Pt-loaded three-dimensionally ordered microporous (3DOM) WO_3_-sensing materials. The addition of Co and Ni led to the formation of more oxygen vacancies on the surface of the WO_3_-sensing material, which resulted in the richer surface adsorption of oxygen [114]. Adding Co and Ni leads to more oxygen vacancies on the WO_3_ surface, resulting in the richer surface adsorption of oxygen. Meanwhile, the addition of Co and Ni narrows the band gap of the sensitive materials. It promotes the increase in electron mobility, which contributes to improving the sensing performance. DOM PtCo-WO_3_ and PtNi-WO_3_ exhibit high selectivity and sensitivity for xylene detection. Among them, the 3DOM PtCo- WO_3_ with atomically dispersed Pt exhibited the most heightened sensitivity of 3.91 ppm^−1^ and the lowest detection limit of 1.08 ppb, and its sensing process is shown in Figure 10e. Researchers can explore more kinds of single atoms interacting with WO_3_ support to enhance gas-sensitive performance.

### 5.3. ZnO-Based Support

**Figure 11:**
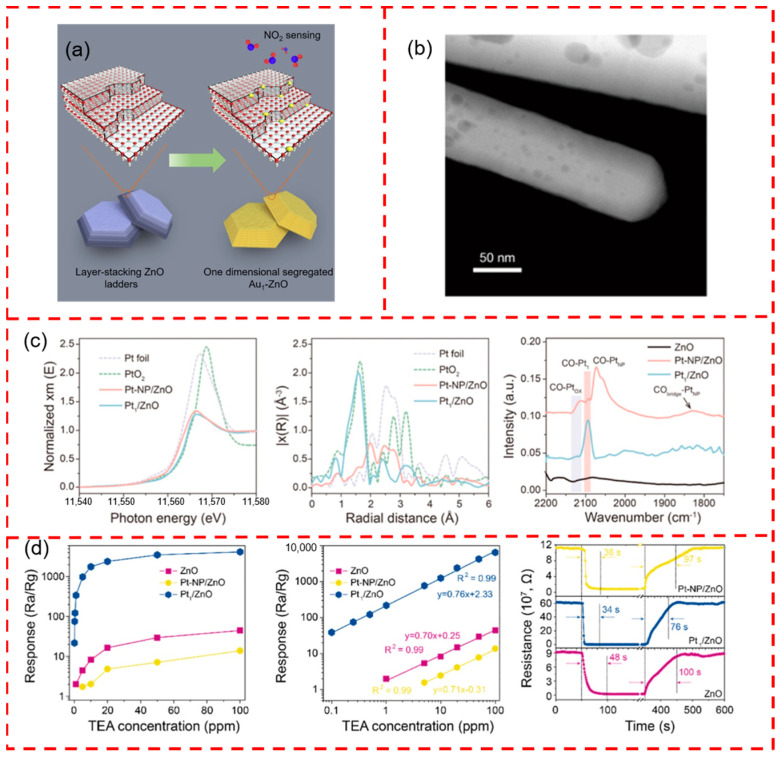
(**a**) Schematic representation of Au single atom anchored at the unsaturated step position of ZnO. Reprinted with permission from Ref. [116]. Copyright 2020, Elsevier. (**b**) TEM image of Pt_1_/ZnO. (**c**) XANES spectrograms of ZnO, Pt-NP/ZnO, Pt_1_/ZnO, EXAFS spectra of Pt K-edge, and CO-DRIFTS spectra. (**d**) The plot of response variation in individual sensors of ZnO, Pt-NP/ZnO, and Pt_1_/ZnO in the range of TEA concentration from 0.1 to 100 ppm, linear relationship plot, and recovery of response in 100 ppm TEA. Reprinted with permission from Ref. [117]. Copyright 2023, American Chemical Society.

ZnO, another common n-type metal oxide semiconductor, is also one of the most studied sensitive materials for gas sensors [115]. ZnO has abundant nanostructures, and different nanostructures can provide various anchoring sites for the loading of SACs. For example, Xue et al. developed a freestanding ladder-like ZnO surface with many unsaturated step defects by controlling the temperature and solvent environment of the reaction system (Figure 11a) [116]. DFT theoretical calculations confirmed that the anchoring of Au single atoms was more stable at the step positions than at the surface positions. The gas-sensitive properties of Au_1_-ZnO, Au-NP-ZnO, and ZnO were further investigated, and the Au_1_/ZnO gas sensors achieved a 12.6-fold response to 300 ppb NO_2_ at 150 °C, which is 1.6-fold and 7-fold higher than that of the Au-NP-ZnO and ZnO sensors, respectively. The Au single atom promotes the activation reaction between NO_2_ and the adsorbed oxygen by facilitating the interaction between NO_2_ and the electron transfer between the support. Liu et al. anchored Pt single atoms on ZnO nanorods (Figure 11b) to achieve the ultra-sensitive detection of triethylamine gas [117]. Pt-NP/ZnO and Pt_1_/ZnO were characterized and analyzed by XPS and EXAFS, and the binding energy of Pt^2+^ in Pt_1_/ZnO showed a significant blue shift compared to that of Pt-NP/ZnO, suggesting the presence of a charge transfer between single Pt atoms and the surface of ZnO. The Pt_1_/ZnO samples had only Pt-O coordination peaks, indicating that the Pt atoms existed in an isolated state. The Pt-NP/ZnO has a characteristic peak of Pt-Pt coordination, reflecting the presence of Pt in the form of clustered nanoparticles (Figure 11c). DRIFTS analysis, using CO as a probe molecule, showed the presence of only 2092 cm^−1^ single bands in the Pt_1_/ZnO sample, demonstrating the presence of Pt^n+^ in the absence of metallic Pt (Figure 11c). The Pt_1_/ZnO gas sensor achieved a 4170-fold response to 100 ppm TEA at 200 °C, which is 92 times that of pure ZnO. Also, the response time (34 s) and recovery time (76 s) were shorter (Figure 11d). DFT calculations showed that the effective adsorption and activation of the TEA molecules on the Pt_1_/ZnO facilitated the interaction between the TEA molecules and the reactive oxygen species, which significantly improved the sensing performance of the Pt_1_/ZnO sensor.

Rong et al. synthesized a porous Ag-LaFeO_3_ @ZnO core-shell sphere structure by isolating the Pt monoatomic precursor with the help of the porous structure of ZIF-8 and removing the ligand from the precursor by subsequent heat treatment [118]. The dispersion of Pt_1-2_ atoms on the porous Ag-LaFeO_3_@ZnO support significantly enhanced the adsorption energy of methanol and oxygen on the surface of the sensitive material and promoted the diffusion, adsorption, and reaction of methanol and oxygen on the surface of Ag-LaFeO_3_@ZnO. The Ag-LaFeO_3_@ZnO-Pt sensor has a response of 453.80 for 5 ppm methanol gas at a lower operating temperature of 86 °C. The study of SAC-functionalized ZnO reveals that the morphology of the material has a great influence on the loading of SAC. This feature can also be applied to other metal oxide materials.

### 5.4. In_2_O_3_-Based Support

**Figure 12:**
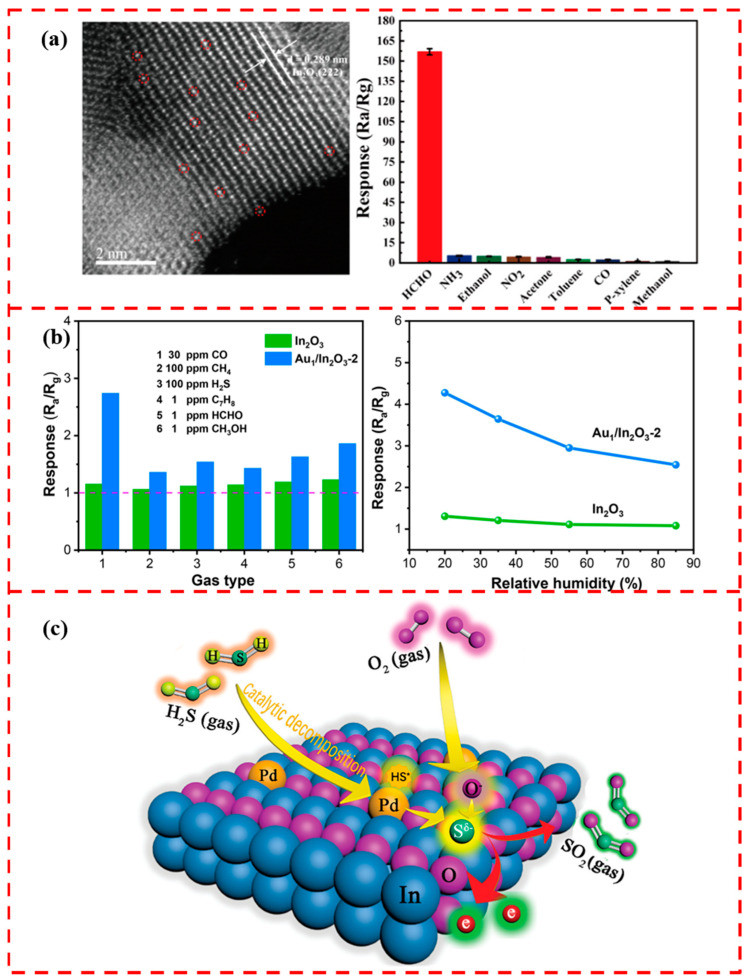
(**a**) HAADF-STEM image of single-atom Au-modified In_2_O_3_, where red circles highlight monodisperse Au atoms and its high selectivity to formaldehyde gas. Reprinted with permission from Ref. [119]. Copyright 2020, American Chemical Society. (**b**) High selectivity and humidity resistance of Au_1_/In_2_O_3_ to CO. Reprinted with permission from Ref. [120]. Copyright 2023, Elsevier. (**c**) Schematic diagram of Pd single-atom-loaded In_2_O_3_ sensing to H_2_S gas. Reprinted with permission from Ref. [56]. Copyright 2021, John Wiley and Sons.

Several scholars have investigated the gas-sensitive properties of In_2_O_3_ modified with single atoms of noble metals Au, Pt, and Pd. Gu et al. loaded atomically dispersed Au on In_2_O_3_ nanosheets by the ultraviolet (UV)-assisted reduction method [119]. In situ, CO-DRIFTS, XPS, and other characterization techniques confirmed the successful loading of Au single atoms. In a series of samples with different Au loadings, 0.25% Au/In_2_O_3_ achieved a maximum response value of 85.67 in formaldehyde gas at 100 °C at 50 ppm. The atomically dispersed Au reduces the activation energy required for the reaction and increases the number of reactive sites. The homogeneity of the single-atom active sites resulted in a higher selectivity of the sensitive material for the adsorption of formaldehyde (Figure 12a). Li et al. successfully loaded Au single atoms on the surface of In_2_O_3_ porous nanospheres for the rapid detection of CO [120]. The XPS results show that single-atom Au interacts with the support and therefore exists in an ionic state. In the detection of reducing gases such as CO, CH_4_, H_2_S, and C_7_H_8_, pure In_2_O_3_ showed poor selectivity for CO. The Au single-atom-loaded Au_1_/In_2_O_3_-2 sensor exhibited significantly enhanced selectivity for CO (Figure 12b). The selectivity to CO demonstrates the potential of Au_1_/In_2_O_3_-2 for application in coal mining environments.

The valence state of single atoms loaded on the surface of In_2_O_3_ also affects the gas-sensitive properties of the material to a great extent. Gu et al. investigated the sensing properties of Pt-loaded In_2_O_3_ nanosheets for triethylamine before and after hydrogen treatment [121]. HAADF-STEM, EXAFS, and XANES characterizations confirmed that the Pt on the surface of 0.25 Pt/In_2_O_3_ samples was highly dispersed before and after hydrogen treatment. In the gas-sensitive performance test of TEA, the hydrogen-treated 0.25 Pt/In_2_O_3_ (H_2_) sensor had a lower operating temperature and higher response value than the non-hydrogen-treated 0.25 Pt/In_2_O_3_ sensor at the same test concentration. The analysis of the XPS results showed a higher adsorbed oxygen content on the surface of the In_2_O_3_ after hydrogen treatment. The lower valence state of surface-loaded Pt is more favorable for adsorbed oxygen generation. Pt loading and hydrogen treatment can improve the electron mobility of In_2_O_3_. The increase in the amount of low-valence Pt after hydrogen treatment promotes the adsorbed oxygen generation. It lowers the TEA response energy barrier, thus increasing the sensitivity of the material to TEA.

Liu et al. took advantage of the porous and surface functional group-rich nature of In-MOFs as a scaffold to support Pd precursor ions [56]. This metal–organic framework template method was employed to synthesize Pd single-atom-loaded In_2_O_3_ with the increase in Pd loading, and Pd single atoms aggregated into PdO nanoparticles. In detecting H_2_S gas, the Pd single-atom-loaded In_2_O_3_ showed the highest response to H_2_S with high selectivity compared to Pd nanoparticles and PdO nanoparticle-loaded In_2_O_3_ sensors. The Pd single atom has an oxidation state different from other forms of Pd and exists as a positive valence state on the surface of In_2_O_3_. This special valence state makes the Pd single atom more active. The Pd single-atom catalysts were able to significantly reduce the potential barrier of the H_2_S gas decomposition reaction, which increased the number of electrons transferred from the H_2_S molecule to the active site of the Pd atom, the sensing process of which is shown in Figure 12c. This indicates that the Pd single-atom catalyst can effectively change the kinetic process of the gas-sensitive reaction. More kinds of single-atom interactions with In_2_O_3_ are expected to be further explored by researchers.

### 5.5. Other Metal Oxides-Based Supports

**Figure 13:**
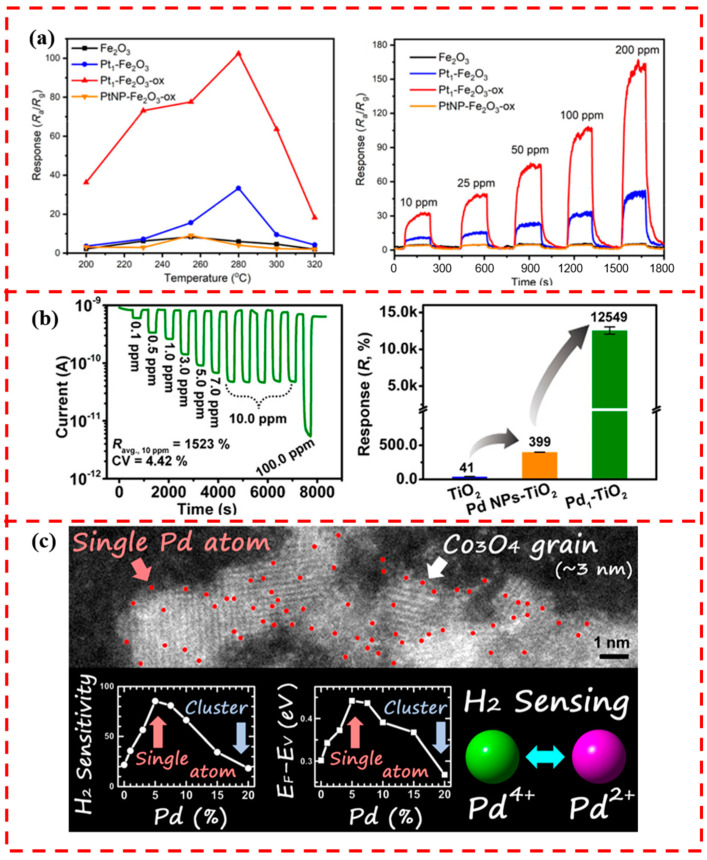
(**a**) Response of Fe_2_O_3_, Pt_1_-Fe_2_O_3_, and Pt_1_-Fe_2_O_3_-ox to 100 ppm C_2_H_5_OH at operating temperature of 200–320 °C to 10–200 ppm concentration of C_2_H_5_OH at 280 °C. Reprinted with permission from Ref. [122]. Copyright 2021, Springer Nature. (**b**) Response curves of Pd_1_-TiO_2_ to CO in the concentration range of 0.1–100 ppm at room temperature and histograms of pristine TiO_2_, Pd-NPs-TiO_2_, and Pd_1_-TiO_2_ nanoflowers to 100 ppm CO. Reprinted with permission from Ref. [123]. Copyright 2021, American Chemical Society. (**c**) Plot of Pd in the form of loading on the surface of Co_3_O_4_ as a function of loading amount. Reprinted with permission from Ref. [124]. Copyright 2020, American Chemical Society.

In the study of metal oxide gas-sensitive materials modified by single-atom catalysts, the SnO_2_, WO_3_, ZnO, and In_2_O_3_ mentioned above are the more studied supports. Some other metal oxide-related reports appeared. For example, Li et al. loaded single-atom Pt on porous γ-Fe_2_O_3_ nanoparticles, achieving an efficient detection of ethanol [122]. The Pt_1_-Fe_2_O_3_-ox sensor with a higher oxidation state Pt exhibited a higher response to ethanol gas (Figure 13a) because the high-valent Pt single atom can effectively increase the adsorption capacity of ethanol gas through charge transfer with an Fe_2_O_3_ support, which improves the gas sensing performance. Ye et al. synthesized Pd single-atom-modified TiO_2_ nanoflowers by the photochemical method for CO gas sensing [123]. In situ DRIFT characterizations showed that Pd_1_-TiO_2_ had the strongest adsorption capacity for CO at the same exposure time, higher than Pd-NPs-TiO_2_ and TiO_2_. Five individually fabricated Pd_1_-TiO_2_ gas sensors achieved an average response of (12,549 ± 494)% to 100 ppm CO at room temperature, the highest value of all the reported sensitive materials operating at room temperature (Figure 13b). Meanwhile, the Pd_1_-TiO_2_ showed excellent CO selectivity in 12 common interfering gases. Koga explored the effect of Pd loading on the hydrogen-sensing performance of mesoporous p-type Co_3_O_4_ nanoparticles [124]. The loading of Pd single atoms on the surface of Co_3_O_4_ could reach up to 5%, and a further increase in loading led to the formation of Pd oxide clusters. Pd single atoms existed at +4 valence on the surface of Co_3_O_4_, and after exposure to 1000 ppm H_2_, about 10% of Pd^4+^ was reduced to Pd^2+^. It is suggested that the catalytic redox process from Pd^4+^ to Pd^2+^ accelerated hydrogen sensing, which enhanced the sensitivity and speed of Co_3_O_4_ response to hydrogen gas (Figure 13c). At present, the studies of metal oxide gas-sensitive materials modified by SACs are still scarce, and more kinds of metal oxides combined with SACs with gas-sensitive properties need to be discovered by further research.

## 6. Summary and Perspectives

In this review, we summarize the progress and utilization of SACs in the metal oxide -based gas sensors, involving the synthesis and characterization methods, stabilization strategies, and gas sensing performance of SACs. The sensing performance of SACs and other modification strategies are summarized in Table 1. As for the heterostructured and MOF-derived metal oxide strategies, they may help detect gases at room temperature, while SACs need a relatively high temperature. In comparison with the modification of normal micro-nanostructures, and nanoparticles decoration strategies, the SAC decoration strategy shows some advantages regarding response, response/recovery time, and selectivity. In conclusion, SACs have made achievements in some aspects of gas sensing, such as response, response/recovery time, and selectivity. However, the application of SACs in the field of gas sensitivity is still in the initial stage. There are still some important issues that need to be further explored and researched:

(1) The high surface energy of the single atom tends to migrate and agglomerate on the support surface, resulting in catalyst deactivation. At the same time, single atoms tend to anchor at the defects and functional groups on the support surface, and the number of surface defects or functional groups limits the loading content. Due to the above reasons, SACs on the support surface are prone to agglomerate and deactivate under harsh environments such as high temperatures, and the loading content of single atoms generally does not exceed 1 wt%. Therefore, achieving the high stability and high-loading anchoring of SACs is important. The development of an efficient, stable, and versatile synthesis method for SACs is the key to promoting the application of SACs in gas-sensitive fields, which is the problem.

(2) At present, research on the application of SACs in metal oxide-based gas-sensitive materials mainly focuses on noble metals (Au, Ag, etc.), and research on non-precious metal single-atoms (Cu, Fe, etc.) is still relatively rare. Further exploration into more kinds of metal single atoms combined with different metal oxide substrate materials is expected to show surprising gas-sensitive effects. Secondly, most current studies focus on the impact of one-kind single metal atoms on gas sensing performance. However, using two different single metal atoms to develop bimetallic sites needs further exploration regarding the performance of metal oxide-based gas sensors.

(3) Some theoretical simulations have revealed that SACs enhance the gas-sensitizing performance by improving the charge transfer of interfaces. However, the effect of SACs on gas-sensitive performance varies with different coordination environments and local structures on the support materials, and the sensitization mechanism of SACs needs to be further clarified. The coordination of SACs on the support surface has a certain complexity, and synthesizing single-atom sites with specific local coordination environments and oxidation states is still challenging.

## Figures and Tables

**Table 1:** The sensing performance summary of SAC and other strategies.

Material	Strategy	Response	Conc. [ppm]	Temp. [°C]	Response/Recovery Time [s]	Analytes	Ref.
3D Networks ZnO	Micro-Nanostructures	3.338	1600	25	20/>60	ethanol	[125]
WO_3_/ZnO	Heterostructured	96	100	200	45/1350	Triethylamine	[126]
MOF-NiO/SnO_2_	MOF-Derived	5.48	100	25	56/4	CO	[127]
In_2_O_3_/SnO_2_	Heterostructured	1.8	10	240	80/60	SO_2_	[19]
CuO/In_2_O_3_	Nanoparticles Decoration	231.2	100	140	9/17	HCHO	[128]
Pt NCs@SnO_2_@SiC	Noble Metal Decoration	56	500	300	/	ethanol	[103]
Dy doping In_2_O_3_	Doping	85	100	250	5/248	ethanol	[129]
Ag NCs In_2_O_3_	Noble Metal decoration	35.6	100	270	25/43	ethanol	[130]
Zn doping In_2_S_3_/In_2_O_3_	Doping	45	100	100	11/24	ethanol	[131]
Pt SAs@SnO_2_@SiC	Single-Atom Catalysts	119	500	350	15/20	ethanol	[103]
Pt-In_2_O_3_	Single-Atom Catalysts	750	100	200	2/373	HCHO	[132]
Pt SA-SnO_2_	Single-Atom Catalysts	25	5	175	/	HCHO	[133]
Ni SA/SnO_2_	Single-Atom Catalysts	50	40	250	52/45	SO_2_	[108]
Ag SA-WO_3_	Single-Atom Catalysts	4700	50	175	189/1354	Triethylamine	[113]
Pt SA-ZnO	Single-Atom Catalysts	4170	100	175	34/76	Triethylamine	[117]

## Data Availability

Not applicable.
